# Detecting anaemia at high altitude

**DOI:** 10.1093/emph/eoaa011

**Published:** 2020-04-13

**Authors:** Kaylee Sarna, Gary M Brittenham, Cynthia M Beall

**Affiliations:** e1 Department of Anthropology, Case Western Reserve University, Cleveland, OH 44106, USA; e2 Department of Pediatrics, Columbia University, New York, NY 10027, USA

**Keywords:** altitude, anaemia, Tibetan, adaptation

## ANAEMIA

Anaemia is a pathology of too few red blood cells or too little haemoglobin, the oxygen-carrying molecule in blood, to deliver adequate oxygen for physiological needs [1]. Low oxygen in the air at high altitude also challenges oxygen delivery. Roughly, one-quarter of the global population suffers from anaemia and 1% resides at altitudes above 2500 m [2, 3]. The World Health Organization (WHO) defines anaemia as a haemoglobin concentration <12.0 g/dl among women or 13.0 g/dl among men; it suggests higher cut-offs for populations at altitude [1] WHO adjustments are based on Andean highlanders who have elevated haemoglobin concentration that partly restores oxygen delivery. Because anaemia’s consequences include poorer growth and development and lower work capacity, detection is important. How then should anaemia be detected among highlanders?

## EVOLUTIONARY PERSPECTIVES

Ancient (∼550 my) genetic and molecular oxygen homeostatic pathways regulate haemoglobin concentration in birds, fish, reptiles and mammals. Ethnic Tibetan highlanders have uniquely high frequencies of variant alleles at two loci (EGLN1 and *EPAS1*) encoding proteins regulating those pathways. Hypoxia inducible factors regulate the formation of red blood cells and haemoglobin [4] that offset hypoxia caused by blood loss, nutrient deficiency or high altitude. Most Tibetans have one or two copies of the variants, which increase in frequency with altitude, show signals of natural selection, and are associated with unelevated haemoglobin concentrations [2]. Consequently, Tibetan haemoglobin concentrations—that often lie within the normal sea-level range of variation—are unelevated. Evaluating healthy, iron-sufficient Tibetans using the WHO recommended altitude-adjustment cut-offs overestimates the prevalence of anaemia: for instance, misclassifying as anaemic >75% of healthy ‘iron-sufficient’ Tibetan adults residing at ∼3900 m [5].

## FUTURE IMPLICATIONS

An evolutionary perspective explains that Andean and Tibetan highland populations represent different outcomes of the natural experiment of migration and adaptation to high altitude. Diagnostic criteria derived from one population may not apply to another. In the case of Tibetans such understanding may prevent unnecessary interventions and use of resources. Evidence that Tibetan men and women with elevated haemoglobin concentrations have poorer work capacity [6] and pregnancy outcome [7] suggests that interventions raising haemoglobin concentration above the WHO cut-offs may harm some individuals. To properly identify iron-deficient Tibetan highlanders, appropriate markers of pathological haemoglobin levels are urgently needed. The WHO guidelines for haemoglobin thresholds used to define anaemia are under review [8] and will accommodate, hopefully, the diversity of healthy, iron-sufficient populations with different evolutionary histories.
